# *FADS2* Genetic Variance in Combination with Fatty Acid Intake Might Alter Composition of the Fatty Acids in Brain

**DOI:** 10.1371/journal.pone.0068000

**Published:** 2013-06-27

**Authors:** Thais S. Rizzi, Sophie van der Sluis, Catherine Derom, Evert Thiery, Ronald E. van Kesteren, Nele Jacobs, Sofie Van Gestel, Robert Vlietinck, Matthijs Verhage, Peter Heutink, Danielle Posthuma

**Affiliations:** 1 Department of Clinical Genetics, VU University Medical Centre, Amsterdam, The Netherlands; 2 Department of Functional Genomics, Center for Neurogenomics and Cognitive Research, Neuroscience Campus Amsterdam, VU University Amsterdam, Amsterdam, The Netherlands; 3 Department of Human Genetics, University Hospital Gasthuisberg, Katholieke Universiteit Leuven, Leuven, Belgium; 4 Department of Neurology, Ghent University Hospital, Ghent University, Ghent, Belgium; 5 Department for Psychiatry and Neuropsychology, School for Mental Health and Neuroscience, Maastricht University Medical Centre, Maastricht, The Netherlands; 6 Department of Child and Adolescent Psychiatry, Erasmus University Rotterdam, Rotterdam, The Netherlands; Alexander Flemming Biomedical Sciences Research Center, Greece

## Abstract

Multiple lines of evidence suggest that fatty acids (FA) play an important role in cognitive function. However, little is known about the functional genetic pathways involved in cognition. The main goals of this study were to replicate previously reported interaction effects between breast feeding (BF) and FA desaturase (*FADS*) genetic variation on IQ and to investigate the possible mechanisms by which these variants might moderate BF effect, focusing on brain expression. Using a sample of 534 twins, we observed a trend in the moderation of BF effects on IQ by *FADS2* variation. In addition, we made use of publicly available gene expression databases from both humans (193) and mice (93) and showed that *FADS2* variants also correlate with *FADS1* brain expression (*P*-value<1.1E-03). Our results provide novel clues for the understanding of the genetic mechanisms regulating FA brain expression and improve the current knowledge of the *FADS* moderation effect on cognition.

## Introduction

Dietary fatty acids are increasingly recognized to have important effects on health outcomes and diseases. Specifically, a role of fatty acids has been implicated in depression, schizophrenia, ADHD (Attention Deficit-Hyperactivity Disorder), cardiovascular disease, and cancer amongst other diseases (for review [1], [2]). In addition, previous research showed that variation in fatty acid levels in blood and milk during pregnancy and lactation influences children’s brain development and later on, children’s neurocognitive functioning and academic achievement [3], [4], [5], [6], [7]. Fatty acids affect a number of physiological processes in their role as energy substrates, structural and functional components of cell membranes, precursors of lipid mediators, and components affecting signal transduction pathways and gene transcription [1], [8], [9], [10], [11].

These and other effects of fatty acids are suggested to be mediated to a large extent by the availability of the long-chain poly unsaturated fatty acids (LC-PUFA), including arachidonic acid [ARA; 20∶4(n-6)], docosahexaenoic acid [DHA; 22∶6(n-3)] and eicosapentaenoic acid [EPA; 20∶5(n-3)] [5]. LC-PUFA levels in phospholipids are influenced by diet [12], [13] but can also be produced endogenously, although the latter is less efficient. ARA is typically provided by meat, eggs, and offal, while DHA is found in marine foods, especially in oily sea fish (e.g., herring, mackerel, salmon and tuna). In the absence of oily sea fish consumption, intakes of DHA are very low [14], [15].

**Figure 1:**
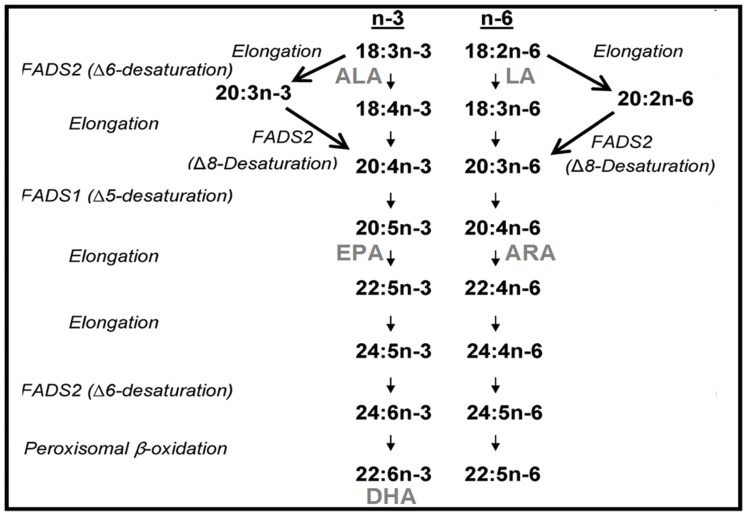
Endogenous pathway for LC-PUFA synthesis DHA, EPA and ARA by enzymatic desaturation and chain elongation steps.

The enzymes FADS1 (fatty acid desaturase 1) and FADS2 (fatty acid desaturase 2) are the rate limiting enzymes in the synthesis of LC-PUFAs ARA, EPA, and DHA from their dietary precursors linoleic acid [LA; 18∶2(n-6)] and a-linolenic acid [ALA; 18∶3(n-3)] [16], [17], [18], [19], [20] (see Figure 1).

Genetic variation in the *FADS1* and *FADS2* genetic cluster on chromosome 11 has been reported to affect the capacity for endogenous formation of LC-PUFAs [21], [22]. Three genetic variants (rs174561, rs3834458, and rs174575) in *FADS1* and *FADS2* have been shown to interfere with the incorporation of n-3 LC-PUFA and DHA from diet in human breast milk [23]. Molto-Puigmarti et al [23] reported decreased DHA levels in plasma phospholipids and breast milk in women who were homozygous for the minor alleles of these variants. The same group also showed that an increase of fish and fish-oil intake does not compensate for the decreased amount of DHA in breast milk in women homozygous for the minor alleles. [23]. These results are consistent with previous reports, in which the same variants in the *FADS1* and *FADS2* genes are associated with less effective desaturated fatty acid production [21], [22]. Located in the same cluster region, *FADS3* shares a high degree of sequence homology with *FADS2* (62%) and *FADS1* (52%), however there is no information on so far about the role of *FADS3* in the fatty acid pathway [24].

Genetic variants in the *FADS2* gene have also been reported to moderate the effect of breast feeding (BF) on cognition [25]. Caspi et al, (2007) showed that breastfed children homozygous for the major allele (C) of rs174575 had a marked increase in IQ of nearly 7.0 points compared to C carriers that were not breastfed, while this effect was absent in children homozygous for the G allele. This result remained significant after ruling out confounding effects such as intrauterine growth differences, maternal cognitive ability, and social class differences [25]. A second *FADS2* variant tested by Caspi et al (rs1535) modified the association between BF and IQ in only one cohort (*P*-value* = *0.01), with children homozygous for the A allele demonstrating an advantageous effect of BF. However, a later study by Steer et al (2010) in nearly 6000 children, did not replicate these findings [26], In contrast to Caspi et al study [25], children homozygous for the G allele exhibited the greatest difference between formula and breastfed such that breastfed children performed similarly irrespective of child genotype whereas formula fed GG children performed worse than other children on formula milk [26]. Both studies reported a large BF effect for rs174575 genotypes, however they reported different causal allele, leaving the moderation effects of rs174575 genotype inconclusive [26].

In spite of these opposing directional effects, multiple lines of evidence do suggest that PUFA levels play an important role in cognitive functioning. As yet, very little is known about how genetic variation in *FADS* genes and subsequent levels of LC-PUFAs may ultimately affect cognitive function. The main goal of this study was to obtain more insight into whether and how genetic variation in the *FADS* gene cluster influences fatty acid metabolism in brain and cognition. To this end, we conducted two studies. First, we tested the moderating relations between genetic variants in the *FADS2* gene, BF and IQ using a sample of 534 twins from the East Flanders Prospective Twins Study. Second, we explored the effects of genetic variation in the *FADS* cluster region (chr11∶61313134-61421211, NCBI36/hg18) on *FADS* brain expression using publicly available genetic databases on brain expression in human and mice. This latter study was performed to understand how genetic variants located in *FADS* cluster region affect expression of the *FADS* cluster genes and IQ. Once we identify the *FADS* genetic variants that affect fatty acid expression levels in brain we will have a better picture of how the interaction between BF and genetic variants affect IQ.

## Materials and Methods

### Ethics

All parents gave their written informed consent according to the local ethics committee guidelines. This project was approved by the Committee of Medical Ethics of the University of Leuven in Belgium.

### Study 1: Does Genetic Variation in FADS2 Moderate the Effect of BF on IQ?

To evaluate the relations between *FADS2* genotype, BF, and IQ we made use of the East Flanders Prospective Twins Survey (EFPTS) data. The EFPTS is a population-based register of twins in the province of East Flanders, Belgium [27]. Data on IQ, BF and DNA were available, as well as data on putative confounders, such as paternal and maternal educational attainment level, gestational age, and birth weight.

#### EFPTS study sample

The EFPTS sample consisted of 534 children (50% males) from 274 EFPTS twin families, including 96 monozygotic twins, 164 dizygotic twins, and 14 single twins. The mean age at time of IQ testing was 10.19 years old (SD = 1.58).

Psychometric IQ was assessed with the Wechsler Intelligence Scales for Children Revised [28]. Scores on 12 subtests of the WISC-R (Information, Similarities, Arithmetic, Vocabulary, Comprehension, Picture Completion, Picture Arrangement, Block Design, Object Assembly, Mazes, Coding, and Digit span) were combined following the WISC manual to obtain an overall total IQ score. The children’s total IQ scores ranged from 64 to 150 (M = 104.59, SD = 14.12). The heritability of the WISC-R IQ-scores was 75% (*P*-value<0.0001), which is in line with previously reported heritability estimates for WISC-R scores in this age group [29], [30].

Information on BF and parental educational level was collected using questionnaires that the parents filled out during their children’s IQ assessment. BF was recorded as a dichotomy (yes/no BF). In this sample, 42% of the children were breastfed.

Both paternal and maternal educational attainment were assessed, and 4 levels were distinguished: primary and lower secondary education (1), higher secondary education (2), higher vocational education (3), and (post) university education (4). Frequencies of educational levels 1 to 4 were 44, 99, 77 and 47 in fathers, and 27, 111, 96 and 33 in mothers, respectively.

Information on gestational age (in weeks) and birth weight (in grams) was obtained from the twins’ medical dossiers recorded at birth. Mean gestational age was 36.70 weeks (SD = 2.36), and mean birth weight was 2502.85 grams (SD = 510.21). This sample size (534) is sufficient to detect SNPs (Single Nucleotide Polymorphisms) explaining 1% of the IQ variance, given a Bonferroni corrected significance level of 0.05.

#### Genotyping

EFPTS genomic DNA was extracted from buccal swabs and placenta using Wizard SV Promega kit (Promega Corporation, Madison, USA). DNA extracted from placental tissue was tested for maternal/sibling contamination using zygosity markers genotyped in the DNA extracted from buccal swabs from twins. Zygosity markers were assessed using the PowerPlex® 16 System kit by Promega. TaqMan SNP Genotyping Assays® commercial protocol and reagents (Taqman*®*, PE Applied Biosystems, Foster city, CA, USA) were used for genotyping genetic variation in the *FADS* cluster region (rs174575 and rs1535, https://www.lifetechnologies.com/global/en/home.html). Each TaqMan probes contains fluorophore and quencher molecules at the 5′ end of each probe. We used VIC or FAM reporter dyes which were linked to the 5′ end of each allele of each genetic variant. In addition at the 3′ end a nonfluorescent quencher (NFQ) and a minor grove binder (MGB) were added to measure reporter dye contributions more accurately and to increase melting temperature without increasing probe length. Deviation from Hardy-Weinberg equilibrium for all genotyped markers was tested using PLINK [31].

#### Statistical analyses

Main effects of genotype and BF, and interaction effects between genotype and BF, on age-and-sex-corrected IQ scores were tested using linear regression. All statistical analyses were carried out in QTDT [32], which accommodates the familial structure of the data. Following the Caspi et al [25] and Steer et al [26] studies, we tested whether rs174575 and rs1535 moderate the effect of BF on later IQ. All analyses included a correction for possible confounding effects of parental education, gestational age, and birth weight.

### Study 2: Do Genetic Variants in FADS2 Affect FADS Brain Expression?

To understand how genetic variants moderates BF effect on IQ, we tested if *FADS* genetic variants regulates fatty acid expression levels in brain. We used publicly available genotypic and brain expression data of human donors [33] and mouse data [34]. In this second study we investigated whether genetic variants in the *FADS* cluster region affect *FADS1*, *FADS2* and *FADS3* brain expression. In addition we also look if these genetic variants might alter binding of transcription factors (TF) in the *FADS* cluster region using inslico approach.

#### Human brain expression data

Brain cortex samples were available from 193 individuals of European descent with age of death ≥65 years with no clinical history of stroke, cerebrovascular disease or Lewy bodies [33]. All 193 samples were genotyped using Affymetrix GeneChip Human Mapping 500 K and the expression analysis was done using Illumina HumanRefseq-8 Expression BeadChip [33]. Genotype and expression data of this sample was obtained accessing Amanda Meyers laboratory website (http://labs.med.miami.edu/myers/LFuN/LFuN.html). This sample size (193) is sufficient to detect SNPs (Single Nucleotide Polymorphisms) explaining 8.3% of the expression variance, given a Bonferroni corrected significance level of 0.0011 (total of 43 tested SNPs).

For all 193 individuals obtained from the Myers’ dataset, genomic coverage in the *FADS* cluster area was increased by using the imputation approach implemented in MACH [35]. MACH imputes genotypes of SNPs that are not directly genotyped in the dataset, but that are present on a reference panel. The reference panel used was HapMap II phased data (NCBI build 36 (UCSC hg18)). Genomic coverage of the candidate regions was extended to ∼ 1.5 Mb around the *FADS* cluster area (chr11∶59813134-59813134, NCBI36/hg18). For the brain expression phenotype we made use of the available mRNA intensity information of the genes inside *FADS* cluster region [*FADS1* (NM_013402), *FADS2* (NM_004265) and *FADS3* (NM_021727)].

Genetic association of imputed genotypes for all 193 individuals from the Myers’ dataset was carried out using a weighted linear regression analysis implemented in MACH2QTL [35]. We included imputed SNPs with MAF >0.05, R^2^>0.3 with the reference allele and additionally a quality imputation score >0.90. Genotyped SNPs were selected if genotyping rate was above 90% and excluded if not in Hardy-Weinberg equilibrium (*P*-value <1e-6). The genetic association analysis was carried out in two steps. First we tested the candidate SNPs (rs1535 and rs174575) from Caspi et al [25]. Given their location and previous findings we hypothesized that variation of these SNPs (or functional variants in high LD with them) interfere with *FADS* cluster expression in human brain. For that reason in the second step we tested all imputed and genotyped SNPs inside *FADS* cluster region.

#### Mouse brain expression data

Brain expression analysis was also performed in mice, to test whether and how genetic variants in the *FADS* cluster region play a role in *FADS* expression in the brain of other organism that is often used as animal model to study human complex traits. We used the mice data available in the Genenetwork database (http://www.genenetwork.org/webqtl/main.py). This database contains both genotypic and phenotypic information from different strains of mice, including C57BL/6, DBA/2 and BXD recombinant inbred strains (from BXD1 through BXD100). These BXD inbred mouse strains were generated by crossing C57BL/6J and DBA/2J mice, intercrossing progeny for ten generations with intentional avoidance of sib mating, and finally inbreeding the advance intercross stock for more than 20 generations [36], making the use of BXD and parental strains suitable for quantitative trait studies. Brain expression data was selected from mice studies in which age differences between mice within the same group did not exceed 1 month, testing a total of two brain regions (whole brain and prefrontal cortex) from two different datasets [37], [38]. In addition we selected genetic variants around the *FADS* cluster area (chr19∶10087063-10277170, NCBI36/mm8) of BXD recombinant inbred strains and C57BL/6J and DBA/2J parental strains [39]. Genotype and brain expression information for all BXD strains were download using gene network website (http://www.genenetwork.org/webqtl/main.py).

To test if the genetic variants in *FADS* cluster region are associated with mice brain expression we conducted a genetic linear regression association analysis using Plink [31]. We tested a total of 93 independent mice strains with both genotypic and brain expression information. Analysis was done for whole brain and prefrontal cortex regions separately.

#### Genetic variants and transcription factors binding sites in FADS cluster regions

To investigate if the genetic variants might alter binding of the transcription factors in the *FADS* region we used JASPAR [40] and consequently interfere with *FADS* expression. To test if the selected variants might alter binding site, we used the web interface for an online sequence analysis of regulatory regions presented in the 500 bp surrounded region of our genetic variants. The transcription binding site models for each sequence were selected if the scoring matrices were above 80%. The analysis was done for each allele separately.

## Results

### Study 1: Does Genetic Variation in FADS2 Moderate the Effect of BF on IQ?

For this study we genotyped 534 children from EFPTS population and tested whether the genetic variants moderate the effect of BF on IQ. Hardy Weinberg equilibrium was tested in a subset of unrelated individuals (N = 267) and did not significantly deviate from expected (rs174575 *P*-value = 0.43; rs1535 *P*-value = 0.30). There was no indication of stratification effects on any of these SNPs using the population stratification option in QTDT (rs174575 *P*-value = 0.19; rs1535 *P*-value = 0.25). The minor allele frequencies in this population were 0.35 for rs1535 and 0.25 for rs174575, which is similar with Caspi et al (rs1535 = 0.37 and rs174575 = 0.28) and Steer et al (rs1535 = 0.33 and rs174575 = 0.26) observations [25], [26].

The two genotyped SNPs (rs1535 and rs174575) are located in the first intron of *FADS2* and are in high Linkage Disequilibrium (LD) with each other (D’ = 0.987 and r^2^ = 0.595) and in strong LD with other SNPs throughout the promoter and intragenic regions of *FADS1* and *FADS2* (Figure S1).

**Figure 2:**
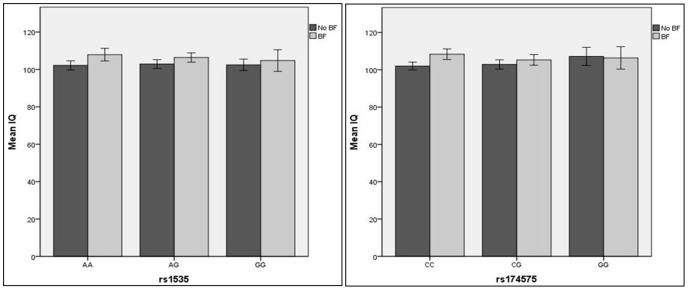
Unadjusted means for Full-scale IQ at 10 years for BF and child *FADS2* rs1535 and rs174575 genotypes. Error bars denote +/−2 Standard Errors of the mean.

Mean IQ scores for the separate genotype groups are reported in Table 1. The difference in IQ scores between BF and non-BF children was 6.2 IQ points for the CC genotype, 3.6 IQ points for the CG genotype, and -1.45 IQ points for the GG genotype, respectively. The rs174575 genotype was not significantly related to IQ (*P*-value = 0.06), but BF was (*P*-value = 0.005), with IQ scores being ∼4.5 IQ points higher in children who were breastfed (mean = 107.21, SD = 14.40) compared to children who did not receive BF (mean = 102.71, SD = 13.59). The 2-sided interaction test between rs174575 and BF was not significant (*P*-value = 0.067), however we can see a trend in the direction as reported in the Caspi et al study, that is the effect of BF on IQ scores was larger in children who carry the C-allele (Figure 2). When we included paternal and maternal educational attainment level as covariates in the model as previous study from Steer et al [26], the interaction effect between rs174575 and BF decreased (2-sided interaction test *P*-value = 0.09), as well as the main effect of BF on IQ (2-sided BF effect *P*-value = 0.08).

**Table 1:** Mean (SD) IQ scores, across different genotype and BF groups.

rs174575	CC	CG	GG	C-carriers	Non-C-carriers
**Non-BF**	102.40 (13.91)	102.32 (13.42)	107.18 (11.35)	102.37 (13.71)	107.18 (11.35)
	N = 181	N = 108	N = 22	N = 289	N = 22
**BF**	108.62 (15.14)	105.91 (14.01)	106.00 (11.65)	107.28 (14.62)	106.00 (11.65)
	N = 106	N = 104	N = 13	N = 210	N = 13
**rs1535**	**AA**	**AG**	**GG**	**A-carriers**	**Non_A_carriers**
**Non-BF**	102.52 (14.08)	102.86 (14.06)	102.82 (10.58)	102.69 (14.04)	102.82 (10.58)
	N = 132	N = 135	N = 44	N = 267	N = 44
**BF**	108.84 (15.64)	106.65 (13.30)	104.75 (15.30)	107.56 (14.32)	104.75(15.3)
	N = 81	N = 114	N = 28	N = 195	N = 28

The two-sided interaction between rs1535 and BF was not significant (*P*-value = 0.12), although the direction of the effect was the same as reported by Caspi et al: the effect of BF was larger in A-allele carriers compared to the homozygous GG group. The difference in IQ scores between BF and non-BF children was 6.3 IQ points for the AA genotype, 3.8 IQ points for the AG genotype, and 1.9 IQ points for the GG genotype, respectively. The rs1535 genotype was not significantly related to IQ (*P*-value = 0.062). When paternal and maternal educational attainment levels were included as covariates in the model, the interaction effect between rs1535 and BF decreased (*P*-value = 0.20).

In addition we investigated whether *FADS2* genotype is related to the exposure to BF (gene-environment correlation), and whether *FADS2* genotype is related to gestational age and birth weight. As both shorter gestational age and lower birth weight are related to lower cognitive functioning [41], [42], a rs174575-BF interaction may be blurred when children with the GG genotype differed from C-carriers in their intrauterine growth. Because the subjects in our sample could not be considered independent observations, these two checks were done using the data of the first twins of each pair only, resulting in a data set of 267 unrelated individuals. These checks showed that children’s genotype was not related to exposure to BF (N = 267, *P*-value = 0.18), and that genotype was not significantly related to gestational age (N = 242, *P*-value = 0.12) or to birth weight (N = 267, *P*-value = 0.06).

In sum, we found no evidence for a moderation effect of rs174575 on BF effects on children’s later IQ score, although we did see a trend in the same direction as reported by Caspi et al The main effect of BF was dependent on the educational level of the parents; highly educated mothers were more likely to breastfeed and their children generally scored better on the IQ test. After including paternal and maternal education as covariates in our model, the main effect of BF on IQ was no longer significant.

### Second Study: FADS Brain Expression

To investigate which genetic variants influence *FADS* brain expression and how they might influence RNA expression, we made use of two datasets, one human [33] and one rodent [37], [38]. Human *FADS1* and *FADS2* both encode proteins of 444 amino acids and share 61% nucleotide sequence identity. In addition, the *FADS2* cDNA extends approximately 140 nt upstream in the 5′ untranslated region (UTR). *FADS3* encodes a 445 amino acid and shares sequence identities with *FADS1* and *FADS2* of 52% and 62% respectively [20]. Alignments of amino acid sequences of *FADS1, FADS2* and *FADS3* with homologues mouse genes reveal strong evolutionary conservation with sequence identities of up to 90% (Figure S2). Testing genetic variants inside *FADS* cluster region in both humans and mice might help unveiling genetic functional mechanisms of *FA* metabolism in the brain.

All genotyped genetic variants located in the *FADS* cluster human region (chr11: chr11∶61313134-61421211,NCBI36/hg18) were tested for association with RNA brain expression of *FADS1* (Accession number NM_013402), *FADS2* (Accession number NM_004265) or *FADS3* (Accession number NM_021727).

We started our analysis with Caspi et al candidate SNPs (rs1535 and rs174575) [25]. In our study, rs174575, which was previously implicated in intelligence in the Caspi et al study [25], was significantly associated with *FADS1* human brain expression as shown in Tables 2 and 3 (*P*-value <0.01, a total of 5 tests).This result suggests that rs174575 might moderate BF effect on IQ via expression of the *FADS1* or at least is in linkage disequilibrium with the functional variant(s) that moderate(s) the BF effect.

**Table 2:** Association of the candidate variants, previous implicated in intelligence, with the human brain expression of *FADS1*, *FADS2* and *FADS3.*

*FADS1* brain expression					
Chromosome	Name	Position	Reference allele	Effect (beta)	P
11	rs1535	61354548	G	−73.65	1.1E-05
11	rs174575	61358579	G	−57.343	1.9E-03
***FADS2*** ** brain expression**					
**Chromosome**	**Name**	**Position**	**Reference allele**	**Effect (beta)**	**P**
11	rs1535	61354548	G	−61.029	0.01409
11	rs174575	61358579	G	−47.196	0.08449
***FADS3*** ** brain expression**					
**Chromosome**	**Name**	**Position**	**Reference allele**	**Effect (beta)**	**P**
11	rs1535	61354548	G	11.414	0.06633
11	rs174575	61358579	G	8.85	0.1958

**Table 3:** Means (SD) of human brain expression scores, differentiated across genotype of the candidate variants from Table 2.

rs174575	CC	CG	GG
***FADS1***	407.2 (179.4)	326.6 (82.47)	346.9 (139.5)
	N = 109	N = 71	N = 11
***FADS2***	568.8 (261.8)	475.6 (153.3)	576.7 (189.4)
	N = 109	N = 71	N = 11
***FADS3***	210.6 (54.07)	216 (52.06)	231.2 (66.94)
	N = 108	N = 71	N = 11
**rs1535**	**AA**	**AG**	**GG**
***FADS1***	431.2 (188.2)	328.3 (87.03)	322.2 (118.5)
	N = 86	N = 86	N = 20
***FADS2***	591.6 (275.3)	478 (162.7)	529.6 (171.5)
	N = 86	N = 86	N = 20
***FADS3***	208.6 (52.78)	216.6 (54.57)	227.4 (57.61)
	N = 85	N = 86	N = 20

From all 43 tested SNPs in human *FADS* cluster region, 16 were associated with levels of *FADS1* human brain expression after Bonferroni correction for multiple testing (*P*-value<1.1E-03, total of 43 tested SNPs), including 2 SNPs (rs174583 and rs174556) located in intronic regions of *FADS2* and *FADS1,* that were previously reported to be associated with fatty acid composition in human blood [21], [22] (Table 4).

**Table 4:** Association of all genetic variants present in *FADS* clusters region and *FADS1* human brain expression.

Chromosome	Name	Position	Referenceallele	Effect(beta)	P
11	rs102275	61314379	T	72.851	0.000014
11	rs174538	61316657	G	70.326	0.000027
11	rs4246215	61320875	T	−64.268	0.000132
11	rs174541	61322484	T	64.467	0.000129
11	rs174545	61325882	G	−72.483	0.000015
11	rs174546	61326406	T	−72.488	0.000015
11	rs174547	61327359	T	72.354	0.000015
11	rs174548	61327924	G	−75.982	0.000016
11	rs174549	61327958	G	76.131	0.000016
11	rs174550	61328054	T	72.866	0.000013
11	rs174555	61336336	T	76.529	0.000015
11	rs174556	61337211	T	−76.576	0.000015
11	rs1535	61354548	G	−73.649	0.000011
11	rs2845573	61358484	G	−79.050	0.006618
11	rs174575	61358579	G	−57.338	0.001907
11	rs2727270	61359813	T	−74.487	0.005342
11	rs2727271	61359934	T	−74.496	0.005348
11	rs174576	61360086	C	73.887	0.000011
11	rs2524299	61361358	T	−74.588	0.005352
11	rs174577	61361390	C	73.936	0.000011
11	rs2072114	61361791	G	−74.788	0.005357
11	rs174579	61362189	T	−56.617	0.002887
11	rs174583	61366326	T	−74.205	0.000011
11	rs174585	61368270	G	59.799	0.002356
11	rs2851682	61372588	G	−81.562	0.007002
11	rs2526678	61380369	G	84.686	0.007752
11	rs174605	61383497	T	−51.862	0.001582
11	rs174611	61384457	T	53.692	0.001262
11	rs174616	61385698	G	40.593	0.009304
11	rs482548	61389758	T	25.131	0.327000
11	rs17764324	61391664	T	−33.948	0.235000
11	rs17831757	61391776	T	32.236	0.257600
11	rs11230815	61392702	G	−31.737	0.264400
11	rs174626	61393633	G	−33.711	0.034350
11	rs7104849	61394620	G	−29.541	0.296200
11	rs472031	61394996	G	−26.181	0.312100
11	rs7482316	61396774	G	−28.832	0.307100
11	rs174449	61396955	G	−43.815	0.006580
11	rs174450	61398118	T	33.926	0.034960
11	rs174634	61403963	G	−33.942	0.080790
11	rs1000778	61411881	G	33.923	0.080990
11	rs174455	61412693	G	−33.010	0.045290
11	rs174456	61412758	C	−33.699	0.082740

A1 is the tested allele.

In addition, we tested whether genetic variants in the *FADS* mice cluster genomic region control *FADS1*, *FADS2* and *FADS3* brain expression. We tested a total of 8 transcripts expressed in two brain regions, whole brain and prefrontal cortex, and 2 genetic variants present in the upstream region of *FADS1* (rs6413006) and intronic region of *FADS2* (D19Mit42) in 93 independent mice strains using linear regression analysis in PLINK [31]. Genetic variants were considered associated with brain expression if significance level bellow 0.007 (total of 2 tested SNPs, 3 genes and 2 brain regions). Brain expression analyses in mice showed that D19Mit42 and rs6413006 genetic variants are associated with the expression of *FADS1* (ID reference 1440444_at) in the whole brain (10–14 weeks of age) (*P*-value<8.5e-05) and with *FADS1* (ID reference 1440444_at) and *FADS2* (ID reference 1419031_at) expression in the prefrontal cortex (8–9 weeks of age) (*P*-value<8e-04) after correcting for multiple tests. In Table 5 we can see the genetic variants and the significant associated trait for each brain region (*P*-values <0.007) (See Table 5).

**Table 5:** List of *FADS* genetic variants significantly associated with *FADS* expression for each mice brain region (*P*-values <0.007).

Brain expression type	Gene expressed	SNP	SNP localization	Bp position	P	Beta
Whole Brain	*FADS1*	D19Mit42	3 Intron *FADS2*	10150049	8.43E-05	0.182
Whole Brain	*FADS1*	rs6413006	5′ *FADS1*	10241575	8.43E-05	0.182
Prefrontal cortex	*FADS1*	D19Mit42	3 Intron *FADS2*	10150049	3.78E-10	0.3837
Prefrontal cortex	*FADS1*	rs6413006	5′ *FADS1*	10241575	3.78E-10	0.3837
Prefrontal cortex	*FADS2*	D19Mit42	3 Intron *FADS2*	10150049	0.000762	0.0765
Prefrontal cortex	*FADS2*	rs6413006	5′ *FADS1*	10241575	0.000762	0.0765

The brain expression results in human and mice suggest that genetic variants in *FADS1* and *FADS2* regions correlate with *FADS1* expression in the whole brain, in both humans and mice. Mice data also suggest that genetic variants in the *FADS* cluster region affect *FADS1* and *FADS2* expression in the prefrontal cortex. We did not find any SNPs that were associated with *FADS3* expression neither in the whole brain nor in the prefrontal cortex (*P*-values all >0.05) (data not shown).

One of the genetic variants tested in mice is located in the 5′ UTR *FADS1* intergenic region (rs6413006). *FADS1* and *FADS2* share the same 5′ UTR region both in mice and humans, however sequence comparison of human and mouse homologues regions using VISTA computational comparative tool, reveals that the *FADS1* and *FADS2* overlapping 5′ UTR region is less conserved (identity ≤70%) (Figure S3, VISTA computational comparative tool) [43], [44]. One possible explanations for these findings is that the human and mouse TF binding sites might not be in the same 5′ UTR regions and in addition there are many cases where regulatory regions do not align nicely unless you allow for gaps. The other possibility is that rs6413006 and D19Mit42 genetic variants are not the functional variants interfering with *FADS1/2* expression but are in high linkage disequilibrium with more conserved functional sites.

Genetic variants presented in the *FADS* cluster region were associated with *FADS1* expression, including previous SNPs involved with intelligence. Binding site insilico analysis of the human *FADS* cluster region revealed that rs174575 is inside predicted zinc finger protein MZF1 (myeloid zinc finger 1) and NFE2L2 (Nuclear factor (erythroid-derived 2)-like 2) binding sites. In addition we found that rs1535 is predicted to be inside ETS1 (v-ets erythroblastosis virus E26 oncogene homolog 1) and GATA2 (GATA binding protein 2) binding sites. Some other genetic variants previously associated with *FADS1* expression were also inside predicted binding site regions (Table 6).

**Table 6:** Schematic representation of human TF binding sites prediction in the surrounding regions of the 16 SNPs associated with *FADS1* brain expression and Caspi et al associated SNP rs174575 [25] using JASPAR (scoring matrices above 80%).

Name	Position	Allele	TFB	SITE
		T	–	–
rs102275	61314379	C	YY1	GC**C**ATA
		G	–	–
rs174538	61316657	A	–	–
		T	GATA2	GGA**T**A
rs4246215	61320875	G	–	–
		T	–	
rs174541	61322484	C	GATA2	GGAT**C**
		G	–	–
rs174545	61325882	C	–	–
		T	–	–
rs174546	61326406	C	–	–
		T	ETS1	CA**T**CCT
rs174547	61327359	C	FOXC1	G**G**TGCGTA
		G	AP1	TGA**C**TGA
rs174548	61327924	C	–	–
		G	–	–
rs174549	61327958	A	GATA3	AGATA**T**
		T	–	–
rs174550	61328054	C	–	–
		T	–	–
rs174555	61336336	C	–	–
		T	YY1	**T**CCATC
rs174556	61337211	C	GATA2	G**G**ATC
		G	ETS1/GATA2	CAT**C**CT/G**G**ATG
rs1535	61354548	A	–	–
		G	MZF1_1–4	TGAG**G**A
rs174575	61358579	C	NFE2L2	ATGGCTGAG**C**A
		C	SPI1	AA**G**AAGT
rs174576	61360086	A	FOXL1	AAAAAA**T**A
		C	–	–
rs174577	61361390	A	BRCA1	AC**A**ACCC
		C	TFAP2A	GCCTGAAG**C**
rs174583	61366326	T	–	–

SITE bold and underscore is the analyzed allele.

## Discussion

Breast milk is rich in ARA and DHA (fatty acid endogenous pathway end products), which are both involved in many neuronal processes, ranging from effects on membrane fluidity to gene expression regulation. The reported short and long term benefits of BF for children are numerous with a positive influence on cognitive development as one of the most consistently reported [45]. It is also known that dietary fat affects gene expression, leading to pronounced changes in metabolism, cell differentiation, and growth [46], [47]. In light of the evidence implicating dietary fat in cognition, understanding the molecular basis for gene expression action on dietary fat composition in brain is critical to understanding its role in human IQ.

In our study we attempted to elucidate parts of a possible molecular mechanism. First we investigated the relation between *FADS2* genetic variants, BF and IQ. We found a main effect of BF on IQ, which was dependent on the educational level of the parents. Mothers with higher IQ and with more education were more likely to breastfeed and their children generally scored higher on the IQ test [48]. After including paternal and maternal education as covariates in our model, the main BF effect on IQ was no longer significant. Steer et al [26] also reported that maternal educational being an important predictor of child IQ (*P*-value<0.001), however the results remain significant after including parental education as covariate.

Although we did not find strong evidence for a moderation effect of BF on the effect of *FADS2* variants on IQ, we did find a trend in the same direction as was previously reported by Caspi et al [25], i.e., the effect of BF on IQ scores was larger in children who carry the C-allele of rs174575, which would have been statistically significant if we would have carried out a one-sided (directional test). We choose not to do so as the original study by Caspi et al and the later study from Steer et al [26] found opposing directions.

The next independent approach was to investigate through which genetic mechanisms *FADS* genetic variants might influence fatty acid composition in the brain, focusing on possible transcription regulation mechanism. In this second study we investigated whether genetic variants in the *FADS* cluster correlate with gene expression in brain. After imputation and analysis of a human brain mRNA expression dataset [33] we saw that genetic variants previously reported to moderate BF effect on IQ (rs174575 and rs1535) [25] and two SNPs (rs174583 and rs174556) previously reported to be associated with fatty acid composition in the blood [21], [22] were associated with *FADS1* brain expression in our study (*P*-value <2.0E-03). The genetic variant rs174583 is in high linkage disequilibrium with the Caspi et al candidate SNP rs1535 (r^2^ = 1), indicating that some functional variants interfering with *FADS1* production in blood might also affect *FADS1* brain expression.

In addition we showed that also in mice two genetic variants (D19Mit42 and rs6413006) in the *FADS* cluster region correlate with *FADS1* and *FADS2* brain expression. It is interesting to note that, in our study, genetic variants inside *FADS2* intronic regions affect *FADS1* whole brain expression both in mice and humans (*P*-values <1.9E-03). We did not see a significant effect of *FADS* cluster genetic variants on *FADS2* or *FADS3* expression neither in humans (*P*-value >0.01) nor in mice in the whole brain (*P*-value>0.05) but we did observe a genetic effect in mice on *FADS2* expression in the prefrontal cortex (*P*-value<0.0008).

These results suggest a strong influence of genetic variants on the activity of the desaturases in brain. One possible mechanism is that genetic variants might alter binding of the transcription factor in the *FADS* cluster region. JASPAR analysis of the genetic variants inside *FADS* cluster region revealed that some of the predicted binding sites are present only when the sequence contains one of the alleles of these genetic variants. In other words, the variants might weaken the consensus sequence, suggesting a possible biological causal effect of these SNPs on *FADS1* expression. A total of 8 genetic variants were in predicted binding sites when one of the alleles was present. From these 8, 4 of them are in high LD with each other (r^2^ = 1), including rs1535, rs102275, rs174577 and rs174583 (data not shown). These findings indicate that *FADS1* brain expression is affected by a combined effect of multiple genetic variants in the *FADS* gene cluster. In addition, some of these variants are located in TF that interact with specific genes through cis-regulatory elements and interface with common components of the transcriptional apparatus. In other words, genetic variants in *FADS2* gene control the expression of *FADS1* gene.

Even though we only observed a trend in the moderation of BF effects on IQ by *FADS2* variation, we believe that in combination with the results from our second study, there is enough reason to investigate the relation between *FADS* cluster genetic variance and *FADS* brain expression in more detail. One of the limitations of our brain expression study is that we do not have diet information and we could not measure directly the effect of the diet on brain expression. In addition we did not investigate the underlying factors that might also interfere with *FADS* expression in the brain, such as genotype and food preference interaction. Although there is some evidence that genetic variants in *FADS* cluster region are the rate limiting factor in the amount of FA in the breast milk for certain genotypes, independent of food consumption [23], nothing is known about the fatty acid expression in the brain and diet intake. Future studies should use behavioral mouse models to investigate diet-genetic interaction effect on gene expression and brain function, from intrauterine developmental phase until adulthood.

In this study we reported that the main effect of BF on IQ is dependent on the educational level of the parents. Parental educational level has been reported to be an important predictor of children’s educational and behavioral outcomes, indicating that highly educated parents have more chance to have a better income [49], [50], [51]. In addition highly educated parents are more likely to pass on genes related to higher IQ as well provide more intellectually stimulating environments [52]. There is evidence that the correlation between the home environment and offspring intelligence is mediated genetically [52], however nothing is known about the moderation effect of family environment (genetic, income, learning environment, diet) and IQ. In summary our results suggest that genetic variants inside *FADS1* and *FADS2* regions control brain expression of *FADS1* and that the genetic variance in combination with food/breast feed intake might alter composition of the fatty acids in brain thereby possibly influencing cognition.

## Supporting Information

Figure S1 **Linkage disequilibrium (LD) in the** ***FADS*** **gene cluster.** LD block representation of genes and genetic variants present in *FADS* cluster region using Haploview [53,54] (HapMap CEU, NCBI36) and UCSC browser (NCBI36/hg18). Genes are represented in blue, blue bars are exons, blue arrows represent transcription direction. Genetic variants in black were reported to be involved with the amount of FA in blood and breast milk, blue reported to be moderators of the association between BF and IQ and red involved in both types of studies. This track plots the logarithm of the odds (LOD score) for linkage disequilibrium between a given variant pair. The color intensity is proportional to the strength of the LD property for the variant pair. White diamonds indicate pairwise D’ values less than 1 with no statistically significant evidence of LD (LOD <2). Light blue diamond’s indicate high D’ values (>0.99) with low statistical significance (LOD <2). Light pink diamonds are present when the statistical significance is high (LOD > = 2) but the D’ value is low (less than 0.5).
(TIF)

Figure S2 **Amino acid sequence alignment of human and mouse** ***FADS1, FADS2*** **and** ***FADS3*** **.** Human amino acid reference sequences *FADS1*: NP_037534.3, *FADS2*: NP_004256.1 and *FADS3*: NP_068373.1. Mouse amino acid reference sequences *FADS1*: NP_666206.1, *FADS2*: NP_062673.1; *FADS3*: NP_068690.3. Identical amino acids are marked by letter code, “+” symbol are “homologous” substitutions, empty spaces are mismatches and “– “ is missing query.
(TIF)

Figure S3 **A visual representation of the** ***FADS1*** **and** ***FADS2*** **5′ UTR shared region of mouse and human genomic alignments (VISTA comparative tool).** The alignment figure shows the human-mouse conservation curves, where dark and light blue boxes represent exons and UTRs respectively. Gene name appears above the track, the arrow points in the direction of the gene. The VISTA curve is calculated as a windowed-average identity score for the alignment. Each “peaks and valleys” graph represents percent conservation between aligned sequences at a given coordinate on the base genome. Regions are classified as “conserved” by analyzing scores for each base pair in the genomic interval, that is “Minimum Conserved Width” (default value 100 bp) and “Conservation Identity” (default value 70%). A region is considered conserved if the conservation over this region is greater than or equal to the “Conservation Identity” and has the minimum length of “Minimum Conserved Width”. Regions of high conservation are colored according to the annotation as exons (dark blue), UTRs (light blue) or non-coding (pink).
(TIF)
