# Supplementary figures and images for: FADS2 Genetic Variance in Combination with Fatty Acid Intake Might Alter Composition of the Fatty Acids in Brain
Source: PLoS One. 2013 Jun 27;8(6):e68000. doi: 10.1371/journal.pone.0068000 (PMC3694926; doi:10.1371/journal.pone.0068000)

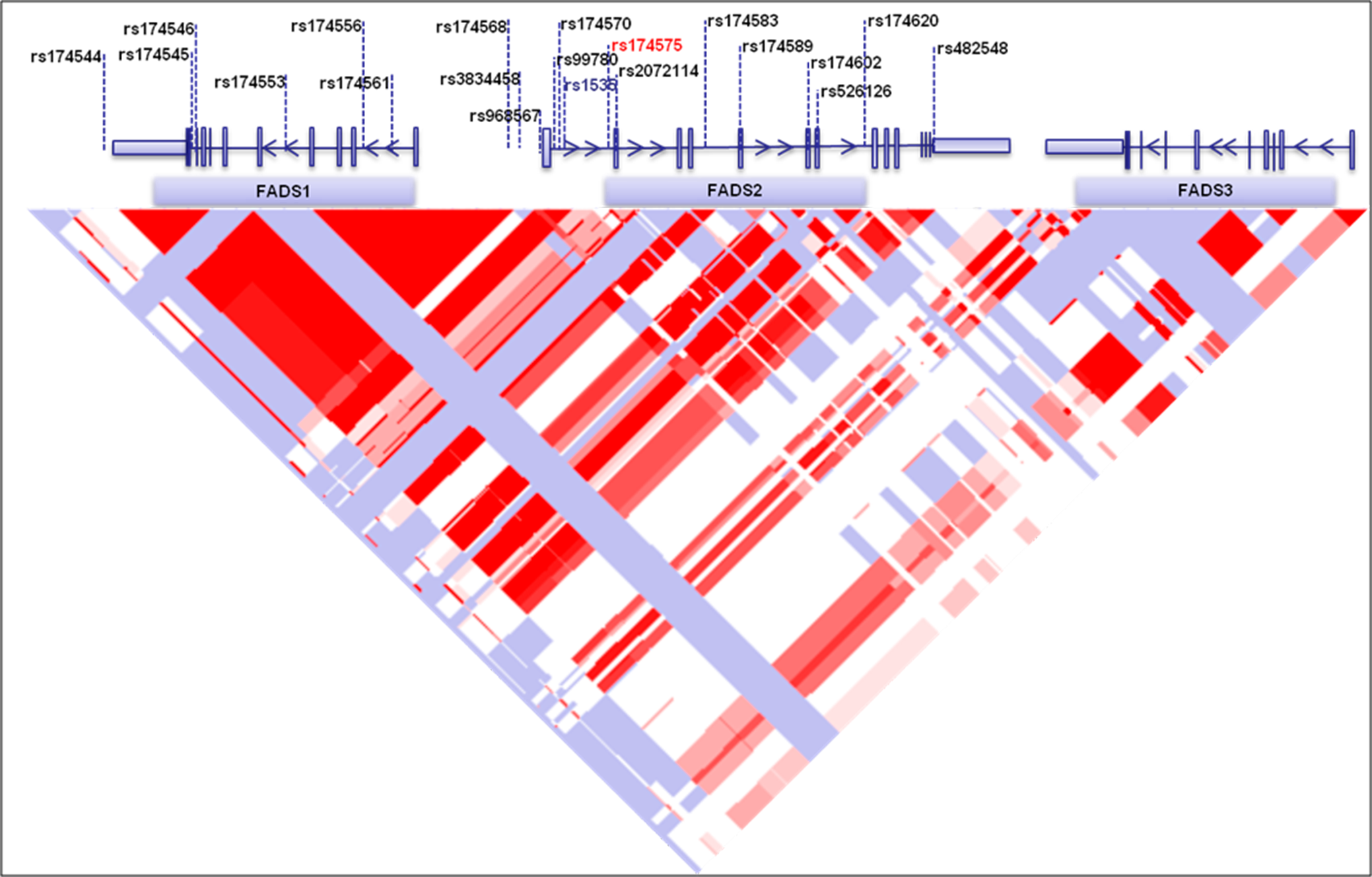

Supplement: Figure S1 — Linkage disequilibrium (LD) in the FADS gene cluster. LD block representation of genes and genetic variants present in FADS cluster region using Haploview [53,54] (HapMap CEU, NCBI36) and UCSC browser (NCBI36/hg18). Genes are represented in blue, blue bars are exons, blue arrows represent transcription direction. Genetic variants in black were reported to be involved with the amount of FA in blood and breast milk, blue reported to be moderators of the association between BF and IQ and red involved in both types of studies. This track plots the logarithm of the odds (LOD score) for linkage disequilibrium between a given variant pair. The color intensity is proportional to the strength of the LD property for the variant pair. White diamonds indicate pairwise D’ values less than 1 with no statistically significant evidence of LD (LOD <2). Light blue diamond’s indicate high D’ values (>0.99) with low statistical significance (LOD <2). Light pink diamonds are present when the statistical significance is high (LOD > = 2) but the D’ value is low (less than 0.5). (TIF) [file pone.0068000.s001.tif]

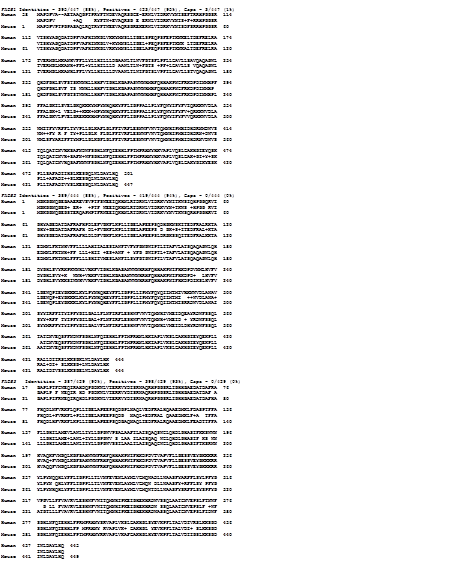

Supplement: Figure S2 — Amino acid sequence alignment of human and mouse FADS1, FADS2 and FADS3 . Human amino acid reference sequences FADS1: NP_037534.3, FADS2: NP_004256.1 and FADS3: NP_068373.1. Mouse amino acid reference sequences FADS1: NP_666206.1, FADS2: NP_062673.1; FADS3: NP_068690.3. Identical amino acids are marked by letter code, “+” symbol are “homologous” substitutions, empty spaces are mismatches and “– “ is missing query. (TIF) [file pone.0068000.s002.tif]

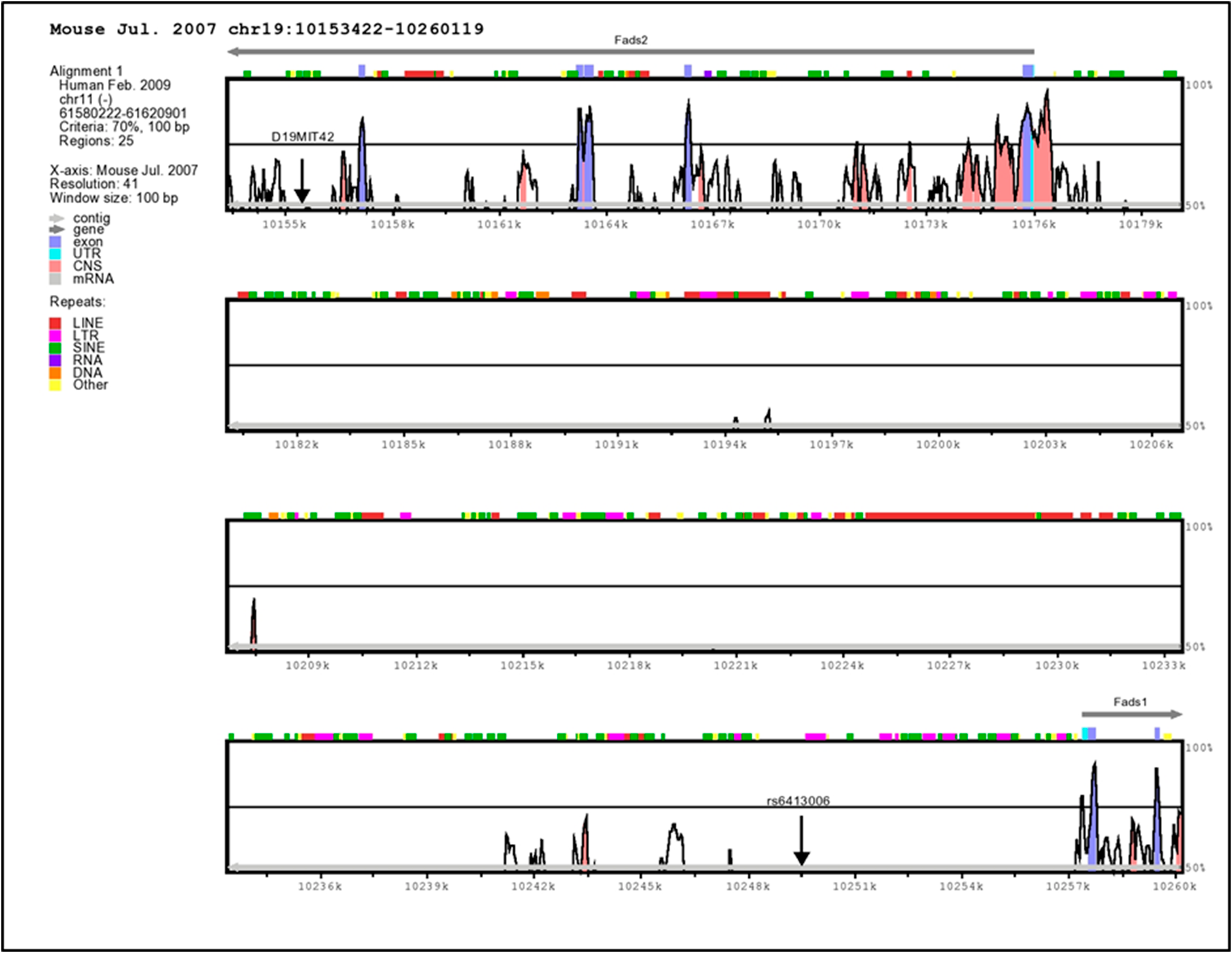

Supplement: Figure S3 — A visual representation of the FADS1 and FADS2 5′ UTR shared region of mouse and human genomic alignments (VISTA comparative tool). The alignment figure shows the human-mouse conservation curves, where dark and light blue boxes represent exons and UTRs respectively. Gene name appears above the track, the arrow points in the direction of the gene. The VISTA curve is calculated as a windowed-average identity score for the alignment. Each “peaks and valleys” graph represents percent conservation between aligned sequences at a given coordinate on the base genome. Regions are classified as “conserved” by analyzing scores for each base pair in the genomic interval, that is “Minimum Conserved Width” (default value 100 bp) and “Conservation Identity” (default value 70%). A region is considered conserved if the conservation over this region is greater than or equal to the “Conservation Identity” and has the minimum length of “Minimum Conserved Width”. Regions of high conservation are colored according to the annotation as exons (dark blue), UTRs (light blue) or non-coding (pink). (TIF) [file pone.0068000.s003.tif]
